# Species Diversity, Abundance, and Habitat Association of Medium- and Large-Sized Mammals in Harego Forest, South Wollo, Ethiopia

**DOI:** 10.1155/sci5/9587584

**Published:** 2024-11-13

**Authors:** Gezahegn Getachew, Dereje Yazezew

**Affiliations:** ^1^Department of Biology, College of Natural Sciences, Wollo University, P. O. Box 1145, Dessie, Ethiopia; ^2^Department of Biology, College of Natural and Computational Sciences, Debre Berhan University, P. O. Box 445, Debre Birhan, Ethiopia

**Keywords:** conservation measures, diversity, endemics, Harego Forest, mammalian species

## Abstract

Despite the importance of the knowledge of mammals' diversity, abundance, and habitat association for designing feasible conservation measures, most of the studies so far are emphasized in protected areas which in turn affects due understanding of the faunal records and conservation endeavors. Hence, we aimed to investigate species diversity, abundance, and habitat association of medium- and large-sized mammals at Harego Forest, South Wollo, Ethiopia, from November 2020 to October 2021, covering both wet and dry seasons. We classified the study area into natural forest, bushland, and *Erica* woodland habitat types based on the topography and vegetation cover. The line transect technique was employed to study the diversity, abundance, and distribution of mammals. The counting of mammals was carried out along 16 transect lines from 06:00 to 10:00 h in the morning and from 15:00 to 18:00 h late in the afternoon when most animals are active in the study area. A total of 260 individual of medium- and large-sized mammals belonging to 15 species, 10 families, and 5 orders were recorded. Among the recorded mammal species, *Theropithecus gelada* was the most abundant species while *Felis serval* was the least in the study area. The abundance of mammals varied significantly among the different habitat types. The highest and lowest mammalian species richness was recorded in the forest and Erica woodland habitats, respectively. The highest species similarity was observed between forest and bushland habitats. The study area is home to different species of mammals including the endemic mammals of Ethiopia that urges conservation stakeholders to design effective conservation measures to sustain the habitat and the mammalian species.

## 1. Introduction

Ethiopia is endowed with unique ecosystems with differences in altitude, climatic conditions, and topographic characteristics from the highest elevations to desert and semidesert ecosystems [1]. The variation in topography and environmental condition are centres of endemism in both the fauna and flora of the country [2]. Thus, Ethiopia has diverse and endemic wildlife species and unique ecosystems [3]. Particularly, the country is endowed with more than 300 mammal species belong to 14 orders, 43 families, 144 genera, and higher endemism (55 mammal species) than other African countries [3–7]. From the total 55 endemic mammalian species, there are 36 rodents, 10 shrews, 3 bats, 2 primates, 2 artiodactyls, 1 carnivore, 1 hare and several unique subspecies of primates and ungulates [5, 8]. Mammals are quite diverse both structurally as well as functionally with more than 5000 extant species recognized throughout the world [9]. In spite of the importance of the knowledge on mammals' diversity, abundance, and habitat association for designing feasible conservation measures, most of the studies are conducted in protected areas [10, 11]. This in turn affects due understanding of the faunal records and conservation endeavor to the country's mammal species [4].

Mammals can successfully colonize diverse habitat types due to diversity in size and morphological, physiological, and behavioral adaptations [12]. Large-sized mammals weigh more than 7 kg while those medium-sized mammals weigh between 2 and 7 kg [7, 11, 13, 14]. Species of large mammals are found on all continents, occurring from the Arctic in the northern hemisphere to the southern tips of the continents and large islands in the southern hemisphere [15]. Africa hosts the highest number and diversity of mammalian species in the world where over 1150 species of mammals belonging to 50 Families are recorded from Africa [16]. The distribution of large mammal species is mainly determined by climate, availability of suitable resources, barriers of dispersal, and inter-specific interaction with those organisms sharing the same area [17, 18]. Animals vary widely in their tolerance to environmental conditions. Hence, some can survive in a variety of habitats, whereas others perish when removed from their natural surroundings [4].

Medium- and large-sized mammals play critical roles in forest ecosystems, acting as key drivers of ecological processes. They contribute for seed dispersal, sustaining in the propagation of plant species, maintaining forest structure and promoting genetic diversity and supporting plant regeneration [19, 20]. In the context of Harego Forest, species such as monkeys likely play significant roles in seed dispersal, particularly for tree species that rely on vertebrate dispersal mechanisms. Mammals also contribute to nutrient cycling and prey–predator dynamics, which help maintain ecosystem stability and resilience allowing ecosystems to recover from disturbances [21, 22]. In Ethiopia's diverse forests, mammals also support forest structure by influencing vegetation composition and promoting biodiversity through their interactions with other species. Understanding their roles provides insights into the functioning and health of these ecosystems and essential for effective conservation.

To understand the potential of an area with regard to the diversity and abundance of animals, the faunal investigation is an important component of research to carryout future conservation action for the animals in an area. In contrary with this principle, the diversity and abundance of medium- and large-sized mammal species of Harego Forest has not been investigated so far. Thus, the lack of comprehensive scientific data on the diversity of mammalian species in Harego Forest urges researchers to conduct this study. Accordingly, this study is basic in bridging the ecological knowledge gap by documenting the species diversity, abundance, and habitat association of medium and large-sized mammals in Harego Forest, northern Ethiopia.

## 2. The Study Area and Methods

### 2.1. The Study Area

**Figure 1:**
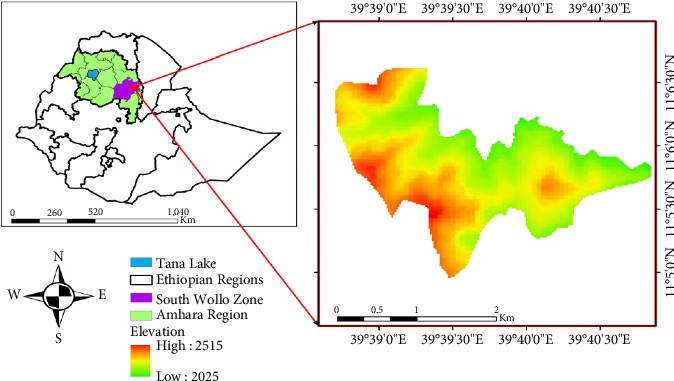
Location map of the study area.

The present study was carried out in Harego Forest, South Wollo Zone, Amhara Regional State, Ethiopia, at a distance of 400 km north of Addis Ababa. The forest is located between two Woredas, namely, Kombolcha to the east and Dessie to the west encircled by human settlements and agricultural fields. The vegetation of the forest is both natural and plantation. The study area lies between 11°03′ 30″–11°08′ 0″ N latitude and 039° 38′ 0″–039°44′ 0″E longitude. The altitude of the area ranges from 1934 to 2526 m asl (Figure 1).

The climate of the Harego Forest is characterized by distinct dry and wet seasons. According to the Kombolcha Meteorological Agency, the rainfall pattern of the area is bimodal with main rainy season (Kiremt or Meher) from June to September and a minor rainy season (Belg) from February to April. The annual rainfall of the study area is over 1200 mm with the highest peak during July (the main rain season). The average monthly temperature of the area is 20°C.

The vegetation type in Harego Forest is characterized by tall and short natural vegetation. High altitudinal locations in the area are covered by *Erica* species, whereas areas at lower altitudinal ranges are covered by tall trees or woody plants. Harego Forest is also home of varieties of mammal species that includes the Menelik's bushbuck (*Tragelaphus scriptus meneliki*), common jackal (*Canis aureus*), spotted hyena (*Crocuta crocuta*), leopard (*Panthera pardus*), Starck's hare (*Lepus starcki*), Ethiopian genet (*Genetta abyssinica*), grivet monkey (*Chlorocebus aethiops*), and gelada (*Theropithecus gelada*).

### 2.2. Methods

Before the actual data collection, a preliminary survey was carried out during the first week of November 2020. In this survey, the study habitats were classified based on the vegetation type and altitudinal ranges. Relevant information about climatic conditions, topography, and biological information of the study area was gathered during this survey.

Based on the topography and vegetation types, the study area was classified as natural forest, bushland, and *Erica* woodland habitats. Data were collected from November 2020 to September 2021, covering both the wet (June to September) and dry seasons (December to March) [23, 24]. The line transect method was used to collect data on medium- and large-sized mammals from the three stratified habitat types. There were sixteen transect lines, which varied in length from 1.3 km to 4.5 km [25] and width ranged from 50 m to 100 depending on the habitat type and topography of the site [26, 27]. We established nine transect lines in the natural forest, four transect lines in the bushland, and other three for *Erica* woodland habitat based on the proportional coverage of the area and landscape types [27].

The counting of the medium- and large-sized mammal populations was conducted by walking on foot at each habitat types. The survey was carried out twice a day, in the morning 06:00–10:00 h and late in the afternoon 15:00–18:00 h to estimate the diversity, abundance, and habitat associations of the mammals when most animals are active in the study area [11, 27]. A total of 60 days were spent to census the mammal species in the study area. Large mammal species identifications and recording were also carried out by using indirect evidences such as feces, footprints, hair samples, calls, territories, burrows, scratches, and other tangible evidences. The indirect method was very useful for surveying naturally rare animals and found at low densities and difficult to record repeatedly, especially for nocturnal species. When mammals were sighted, the number, habitat type, GPS location, and other relevant information were recorded. Identification and recording of large mammals were based on Kingdon [16]. Two survey procedures were used to assess the presence of medium and large mammals; direct observation of animals and indirect evidences including auditory identification and mammalian signs including fresh tracks, faeces, dens, spines/bristles, carcass, and scent marking [28]. Indirect detection indices were very useful when surveying animals such as carnivores that are naturally rare, elusive, found at low densities, and difficult to encounter easily [29].

### 2.3. Data Analysis

The collected data were analyzed by using SPSS software Version 20. We used both descriptive and inferential statistics to present the findings. The Shannon–Wiener Diversity Index was used to calculate and determine the diversity, abundance, and distribution of species between the wet and dry seasons and within habitat types using the following formula [30].

(1) $$\begin{array}{c} H^{\prime }=-\overset{n}{\underset{i}{\sum }}\left(\text{Pi}\right)\left(\text{lnPi}\right), \end{array}$$

where *H*′ = diversity index, ln = natural logarithm, and Pi = proportion of the total sample belonging to the *i*th species (pi = ni/*N*, where ni is the number of individuals in species *i* and *N* is the total number of individuals of all species).

We also used the Shannon–Wiener evenness index (*E*) to determine the mammalian species distribution pattern in the habitats and computed using the formula: *E* = *H*′/*H*_max_, where *H*′ = Shannon–Wiener Diversity Index, and *H*_max_ = lnS, where ln = natural logarithm and *S* = the total number of species in each habitat.

We used Sorenson's coefficient (*S*) to compare the similarity in mammalian species composition between habitats types and computed with the following formula: *S* = 2C/(*S*1 + S2 + S3) [30, 31], where *C* = the number of common species in all three habitats, *S*1 = the number of species in habitat one, *S*2 = the number of species in habitat two, and *S*3 = the number of species in habitat three. We calculated the relative abundance index of species (RAI) by dividing the number of records of each species by the total number of records of all species. Relative abundance = (*n*/*N*) ∗ 100, where *n* is the number of individuals of particular species recorded and N is the total number of individuals of all species in the study area. We used the chi-squared test to determine the significant association of mammal species composition and abundance between different habitats and seasons. The *p* value was set at *p* ≤ 0.05.

## 3. Results

### 3.1. Species Composition of Medium and Large-Sized Mammals

During the present study, a total of 260 individual of medium- and large-sized mammals belonging to 15 species, 10 families, and 5 orders were recorded. These species include eight species of the Order Carnivora, three species of the Order Artiodactyla, two species of the Order Primate, one species of the Order Lagomorpha, and one species of the Order Rodentia (Table 1).

### 3.2. Abundance of Large Mammals

Among the recorded individuals, *T. gelada* was the most abundant species (88, 33.85%), followed by *C. aethiops* (71, 27.31%), and *Felis serval* was the least abundant (2, 0.77%) in the study area (Table 2).

### 3.3. Distribution and Habitat Association of Mammal Species

The highest number of individuals of mammal species were recorded from forest habitat (107, 41.15%), followed by bushland (83, 31.92%) and *Erica* woodland (70, 26.92%) (Table 3). The abundance of mammals among different habitats in the present study area showed statistically significant variations (*t* = 6.93, df = 14, = *p* < 0.001).

The distributions of species varied from habitat to habitat where *S. grimmia*, *O. oreotragus*, *T. gelada*, *C. aethiops*, and *H. cristata* were the most widely distributed species along all habitat types in the study area. *Tragelaphus scriptus meneliki*, *P. pardus*, C. *crocuta*, *C. aureus*, *I. albicauda*, *M. capensis*, and *F. serval* were common in forest and bushland habitats but not observed in *Erica* woodland habitat. *Felis silvestris* was found in bushland and *Erica* woodland habitats. *Lepus starcki* and *G. abyssinica* have restricted distribution and recorded only from *Erica* woodland and forest habitats, respectively (Table 3). Higher diversity and abundance of mammals were recorded during the dry season than that in the wet season.

### 3.4. Species Diversity, Richness, and Similarity Between Habitats

**Figure 2:**
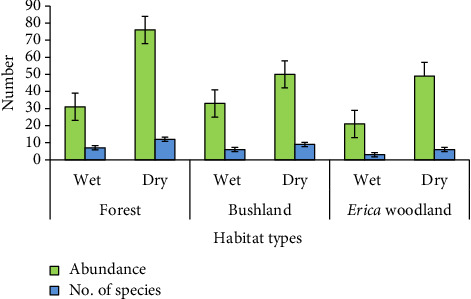
Species diversity and abundance of mammals in different habitat types.

Among the 15 species of large mammals recorded during the present study period, forest habitats had the highest mammal richness (14 species), followed by bushland and *Erica* woodland habitats with 12 and 7 species, respectively (Figure 2). The composition of species in the three habitat types did not show statistically significant variation (*t* = 3.4, df = 2, = *p* > 0.05).

The diversity and evenness of large mammal species varied from habitat to habitat in the present study area. The highest diversity (*H*′ = 1.90) was observed in the forest habitat, followed by bushland (*H*′ = 1.81) and *Erica* woodland (*H*′ = 1.25) (Table 4). Simpson's diversity index was also highest in the forest (*D* = 0.85), followed by bushland habitat (*D* = 0.78), and the lowest was in *Erica* woodland. The highest species evenness was observed in the bushland habitat (*E* = 0.73), followed by forest habitat, and the lowest in the *Erica* woodland habitat (Table 4).

The highest species similarity was observed between forest and bushland (0.92) followed by forest and *Erica* woodland habitats (0.57). The lowest species similarity was observed between bushland and Erica woodland habitats (0.53) during the present study (Table 5).

## 4. Discussion

Documenting the presence or absence of species and monitoring the status of species with conservation concern play great role for ecologists to design future conservation strategies [32]. In this study, we confirmed the existence of a total of 15 species of medium- and large-sized mammals using direct observation and indirect evidences in Harego Forest. Similarly, the same figure (15 mammal species) has been reported by Qufa and Bekele [33] from Lebu Natural Protected Forest, Southwest Shewa, Ethiopia; Kassa and Tekalign [26] from Nech Sar National Park, southern Ethiopia; and Geleta and Bekele [34] from Wacha Protected Forest, western Ethiopia. Other researchers have also reported comparable results. For instance, Girma and Worku [35] confirmed the existence of 16 mammal species in Nensebo Forest, southern Ethiopia; Wale and Yihune [36] identified 18 species of medium- and large-sized mammals at Chimit-Kolla, Abay Gorge, Ethiopia; Abie et al. [7] recorded 20 mammal species in Gibe Sheleko National Park, southern Ethiopia. On the contrary, lower number of medium- and large-sized mammal species (eight species) have been recorded in Humbo Community-Based Forest Area by Lemma and Tekalign [37]; 10 mammal species in Tiski Waterfall, Awi Zone, Ethiopia, by Derebe, Derebe, and Kassaye [27]; and 10 species of mammals at Geremba Mountain Fragment, southern Ethiopia, by Worku and Girma [38]. Higher number of medium- and large-sized mammal species have also been reported in Adaba Community Forest (27 mammals), West Arsi Zone, Ethiopia Bakala and Mekonen [25] and Dati Wolel National Park (28 mammals), Ethiopia, by [11]. The difference observed in the diversity of mammal species in Harego Forest in comparison with other studies possibly attributed to the variability in habitat topography and resource availability, area coverage, vegetation structure and composition, survey period, conservation status of the study area, and intensity of anthropogenic impact.

The present study showed that greater diversity and abundance of medium- and large-sized mammals were recorded from the forest habitat and the lowest numbers of mammals were recorded from the *Erica* woodland habitat. This variation in the abundance of mammals from habitat to habitat might be due to the presence of variations in resource accessibility, habitat cover, and human disturbance in the area [7, 25]. Balakrishinan and Easa [39] revealed that the diversity, distribution, and abundance of mammals are mainly determined by ecological factors such as vegetation composition, floristic structure of the habitat, resource availability, and anthropogenic disturbances. The distribution of species is not uniform throughout their geographical ranges due to the heterogeneity of the natural world that result in an interspersed distribution of habitats of varying quality [25, 40].

This study revealed three of the endemic mammal species of Ethiopia, namely, Starck's hare (*Lepus starcki*), Menelik's bush buck (*Tragelaphus scriptus meneliki*), and gelada (*Theropithecus gelada*). The study also confirmed the existence of one IUCN vulnerable species, leopard (*Panthera pardus*) [7, 41]. The occurrence of endemic and threatened species implies the importance of conservation endeavor to the Harego Forest. *T. s. meneliki* was recorded from bushland and forest habitats but not from *Erica* woodland habitat in Harego forest. This finding is in contrary to the study from Borena Sayint National Park where the species was observed in the riverine forest and *Erica* woodland habitats [42]. Moreover, we confirmed the presence of two primate species, *T. gelada* and *Chlorocebus aethiops*, where *T. gelada* is the most abundant and *C. aethiops* is the second. However, this finding is contrary to the study from Wabe forest fragment where *C. pygerythrus* was the most abundant species, that is, the same genus with *C. aethiops* [43].

Primates ranked first both in distribution and abundance followed by the Order Artiodactyla. This finding is in line with Legese, Bekele, and Youm [43] and in contrary with the observation of Geleta and Bekele [34]. The probable reason for this might be the difference in location and habitat characteristics of the study areas including vegetation structure, composition, cover density, and human encroachment.

The study revealed that highest species similarity was observed between forest and bushland. This finding is in line with Wale and Yihune [36] who reported highest similarity of occurrence of mammal species between natural forest and wooded grassland. The plausible reason for this similarity might be higher forage availability in the two habitats and better cover availability to hide from danger [43]. Moreover, higher diversity and abundance of medium- and large-sized mammals were recorded during the dry season. Other studies also reported similar finding [36, 38]. This might possibly be due to truncated visibility because of the complication of vegetation density and structure during the wet season while there is higher visibility of animals during the dry season as a result of scattered canopy and lower vegetation cover when some plants shed their leaves to prevent water loss.

Harego Forest has several threats including firewood collection, timber, charcoal for the local market, livestock grazing, human encroachment, and road construction across the forest, which were commonly observed in the study area. The forest is the only source of wood for residents around and grazing is allowed in the forest. Similar incidents have been reported from other studies [7, 11, 26, 34, 43] and urgent conservation measure should be launched to avert the situation from becoming worsened in the country.

## 5. Conclusions

This study provides insight into the species diversity, abundance, and habitat association of medium- and large-sized mammals in Harego Forest, South Wollo, Ethiopia. Fifteen mammalian species were confirmed to exist in Harego Forest where the area can be identified as a potential wildlife habitat in which comparably moderate diversity of medium- and large-sized mammal species including the endemics and threatened taxa can be sustainably sheltered. The diversity, distribution, and abundance of medium- and large-sized mammal species in the study area varied because of vegetation types and topographic differences. Although the forest harbors such diversity of mammal species, there are considerable anthropogenic threats posed by surrounding communities. These results emphasize Harego Forest's critical role as a refuge for mammalian biodiversity, reinforcing the importance of sustainable management and conservation actions to mitigate threats like habitat loss and human–wildlife conflict. Therefore, the forest needs strong attention from both the Regional and Federal governments as well as other stakeholders to implement appropriate and feasible wildlife management efforts to sustain these mammal species and the forest. To that end, future studies should aim to monitor population trends and examine the impacts of anthropogenic pressures, thereby supporting adaptive management efforts that can sustain mammal diversity and ecosystem health in the region.

## Figures and Tables

**Table 1:** Mammal species recorded from the Harego forest.

Order	Family	Common name	Scientific name	IUCN conservation status	Method of identification
Artiodactyla	Bovidae	Common duiker	*Sylvicapra grimmia*	LR	Visual
		Klipspringer	*Oreotragus oreotragus*	LR	Visual
		Menelik's bushbuck	*Tragelaphus scriptus meneliki*	LC	Visual

Lagomorpha	Leporidae	Starck's hare	*Lepus starcki*	LC	Visual/feces

Carnivora	Felidae	Leopard	*Panthera pardus*	VU	Visual/feces/sound
		Serval cat	*Felis serval*	LC	Feces/visual
		African wild cat	*Felis silvestris*	LC	Visual
	Hyaenidae	Spotted hyena	*Crocuta crocuta*	LC	Visual/sound/footprint
	Canidae	Common jackal	*Canis aureus*	LC	Visual
	Viverridae	Abyssinian genet	*Genetta abyssinica*	LC	Visual
	Herpestidae	White-tailed mongoose	*Ichneumia albicauda*	LC	Visual
	Mustelidae	Honey badger	*Mellivora capensis*	LC	Visual

Primate	Cercopithecidae	Gelada baboon	*Theropithecus gelada*	LR	Visual
		Grivet monkey	*Chlorocebus aethiops*	LC	Visual

Rodentia	Hystricidae	Crested porcupine	*Histrix cristata*	LC	Spine/visual

**Table 2:** Abundance of medium and large-sized mammals in Harego Forest.

Species name	Abundance	Relative abundance (%)
*Theropithecus gelada*	88	33.85
*Chlorocebus aethiops*	71	27.31
*Crocuta crocuta*	18	6.92
*Sylvicapra grimmia*	17	6.54
*Tragelaphus scriptus meneliki*	13	5.00
*Oreotragus oreotragus*	12	4.62
*Histrix cristata*	9	3.46
*Canis aureus*	6	2.31
*Lepus starcki*	5	1.92
*Panthera pardus*	5	1.92
*Felis silvestris*	4	1.54
*Ichneumia albicauda*	4	1.54
*Genetta abyssinica*	3	1.15
*Mellivora capensis*	3	1.15
*Felis serval*	2	0.77
Total	260	100.00

**Table 3:** Distribution and abundance of mammals in different habitat types.

Species	Habitat types					
	Forest		Bushland		*Erica* woodland	
	Wet	Dry	Wet	Dry	Wet	Dry
*Sylvicapra grimmia*	2	6	1	0	0	3
*Oreotragus oreotragus*	0	7	0	3	2	0
*Tragelaphus scriptus meneliki*	0	8	4	1	0	0
*Lepus starcki*	0	0	0	0	0	5
*Panthera pardus*	3	0	2	0	0	0
*Crocuta Crocuta*	0	12	6	0	0	0
*Canis aureus*	2	2	0	2	0	0
*Genetta abyssinica*	3	0	0	0	0	0
*Felis silvestris*	1	2	0	0	0	1
*Ichneumia albicauda*	0	2	0	2	0	0
*Mellivora capensis*	0	2	0	1	0	0
*Felis serval*	0	1	0	1	0	0
*Theropithecus gelada*	7	12	11	16	13	28
*Chlorocebus aethiops*	14	17	9	17	6	9
*Histrix cristata*	0	4	0	2	0	3
Abundance	31	76	33	50	21	49
Total species	7	12	6	9	3	6
Percentage (%)	41.15		31.92		26.92	

**Table 4:** Species richness and diversity of mammals in different habitats.

Habitats	Number of species	Number of individuals	*H*′	*D*	*H*_max_	*E*
Forest	14	107	1.90	0.85	2.64	0.72
Bushland	12	83	1.81	0.78	2.48	0.73
*Erica* woodland	7	70	1.25	0.59	1.95	0.64

*Note:H*′ = Shannon–Weaver diversity index, *D* = Simpson's Diversity Index, *H*_max_ = natural logarithm of the total number of species, *E* = evenness.

**Table 5:** Species similarity of mammals in different habitat types of the study area.

	Forest	Bushland	*Erica* woodland
Forest	—	0.92	0.57
Bushland	0.92	—	0.53
*Erica* woodland	0.57	0.53	—

## Data Availability

The data that support the findings of this study are available from the corresponding author on request.
